# Sea and Country

**Published:** 1903-07-11

**Authors:** 


### SEA AND COUNTRY.

It not infrequently happens that some members of a family sigh for the sea, whilst others prefer the amusements of the country. It is seldom that the two can be combined by paterfamilias in his endeavour to satisfy all views in selecting a place for the autumn holiday. The new Hotel Imperial, Hythe, Kent, practically does this, for it stands upon the beach facing the sea, and has some thirty acres of gardens at the back, which are well sheltered and most attractive. These gardens have been laid out in lawn-tennis courts and croquet lawns, and are lighted in the evening by electricity. Here lovers of the country may secure absolute quietude, the choice of a variety of shady nooks in which to sit and read, with abundant space for reasonable exercises, and the enjoyment of outdoor amusements. There is an excellent green for bowls, and a large field devoted to archery. The hotel itself is most beautifully equipped, and offers many attractions to visitors. The guests have a private bathing place within a stone's throw of the hotel, and there is a flotilla of boats on the canal behind the gardens which offer lovers of boating several picturesque excursions varying in length from four miles to twenty. The canal abounds in fresh-water fish, especially pike, bream and roach.

Hythe itself has a special charm from the number of trees which it contains, and the neighbourhood is full of historical interest. There is also an eighteen-hole golf-course, which is one of the finest in England.

It was our good fortune to stay a short time at the Hotel Imperial recently, which caused us pleasurable surprise from the infinite variety afforded by the various pleasure facilities which we have already enumerated. We know of few places where a summer holiday can be spent with the prospect of more pleasure and health benefit, and so we venture to direct the attention of our readers to this new and important addition to the attractions offered by English seaside resorts. No doubt a large number of day visitors will be attracted to Hythe by the gardens, and the facilities which they offer for tennis, archery, croquet and bowls. Mr. W. R. Cobay, who has designed and carried out the whole of the decorations and reconstruction of the Hotel Imperial and grounds, may well be proud of his achievement. Visitors to the Cinque Ports and Kentish watering places will be wise to avail themselves of the facilities now afforded at Hythe for a pleasant day's excursion.

At a recent meeting of the South London Cabmen's Association a resolution was unanimously passed expressing horror of bad language especially in public places, and calling upon all cabmen to discountenance and suppress the evil practice.

